# Breeding on the extreme edge: Modulation of the adrenocortical response to acute stress in two High Arctic passerines

**DOI:** 10.1002/jez.1923

**Published:** 2015-03-10

**Authors:** Brian G. Walker, Simone L. Meddle, L. Michael Romero, Meta M. Landys, Jeroen Reneerkens, John C. Wingfield

**Affiliations:** ^1^ Department of BiologyUniversity of WashingtonSeattleWashington; ^2^ Department of BiologyFairfield UniversityFairfieldConnecticut; ^3^ The Roslin Institute & Royal (Dick) School of Veterinary StudiesThe University of EdinburghMidlothianScotlandUK; ^4^ Department of BiologyTufts UniversityMedfordMassachusetts; ^5^ Department of Fisheries and WildlifeOregon State UniversityCorvallisOregon; ^6^ Animal Ecology GroupCentre for Ecological and Evolutionary StudiesUniversity of GroningenGroningenThe Netherlands; ^7^ Department of Neurobiology, Physiology and BehaviorUniversity of CaliforniaDavisCalifornia

## Abstract

Arctic weather in spring is unpredictable and can also be extreme, so Arctic‐breeding birds must be flexible in their breeding to deal with such variability. Unpredictability in weather conditions will only intensify with climate change and this in turn could affect reproductive capability of migratory birds. Adjustments to coping strategies are therefore crucial, so here we examined the plasticity of the adrenocorticotropic stress response in two Arctic songbird species—the snow bunting (*Plectrophenax nivalis*) and Lapland longspur (*Calcarius lapponicus*)—breeding in northwest Greenland. Across the breeding season, the stress response was strongest at arrival and least robust during molt in male snow buntings. Snow bunting females had higher baseline but similar stress‐induced corticosterone levels compared to males. Modification of the stress response was not due to adrenal insensitivity, but likely regulated at the anterior pituitary gland. Compared to independent nestlings and adult snow buntings, parental‐dependent chicks had a more robust stress response. For Lapland longspurs, baseline corticosterone was highest at arrival in both male and females, and arriving males displayed a higher stress response compared to arriving females. Comparison of male corticosterone profiles collected at arrival in Greenland (76°N) and Alaska (67–71°N;) reveal that both species have higher stress responses at the more northern location. Flexibility in the stress response may be typical for birds nesting at the leading edges of their range and this ability will become more relevant as global climate change results in major shifts of breeding habitat and phenology for migratory birds. *J. Exp. Zool. 323A: 266–275, 2015*. © 2015 The Authors. *J. Exp. Zool.* published by Wiley Periodicals, Inc.

Weather conditions in the Arctic environment can be extreme and unpredictable (Walsh et al., 2005; Weatherhead et al., 2010) and it is likely that this variablity and unpredictablity will only increase in the future because of global climate change (Serreze et al., 2000; Post et al., 2001; Sturm et al., 2001 Gaston et al., 2005; Hoye et al., 2007). Indeed, Arctic sea ice is melting earlier in the spring and tundra permafrost is melting to greater depths. The Arctic summer is also getting longer as the tundra and water surfaces take longer to freeze in autumn (Olsson et al., 2003). When migrant songbirds arrive at their northern breeding grounds, they may, in some years, have to cope with inclement weather that can negatively affect breeding. In other years they may be met by weather conditions that are conducive to breeding (Wingfield and Hunt, 2002; Martin and Wiebe, 2004; Wingfield et al., 2004). Determining the mechanisms that migrant birds use to cope with environmental stochasticity and reproduce successfully in the variable Arctic environment, particularly at the very edge of their breeding ranges, remains of great interest.

The songbirds that arrive in spring must be highly flexible to adjust breeding activities in case of inclement weather. They must also be able to take advantage of multiple food resources that are sometimes patchy in their distribution (Martin and Wiebe, 2004; Wingfield et al., 2004). Northern weather conditions on the breeding grounds are typically more severe than those on the wintering grounds and this can result in stimulation of the hypothalamus–pituitary–adrenal (HPA) axis causing an increase in glucocorticoid secretion (Romero et al., 2000; Reneerkens et al., 2002; Meddle et al., 2003). Glucocorticoids (e.g., corticosterone in birds) enable individuals to cope with environmental stressors by enhancing mobilization of energy stores, triggering movements away from the source of stress, and facilitating other facultative behavioral changes (Sapolsky et al., 2000; Romero, 2002; Wingfield and Sapolsky, 2003). However, elevated corticosterone may inhibit reproductive development and delay the onset of breeding despite the need to begin nesting as early as possible in an environment where the window for successful reproduction is only 4–5 weeks (Wingfield and Hunt, 2002; Wingfield and Sapolsky, 2003). Thus, strategies with which to cope with unpredictable environments are critical to ensure appropriate timing of nesting (Martin and Wiebe, 2004). Such mechanisms include modulation of the stress response driven by changes in stress hormone titers and mineralocorticoid and glucocorticoid receptor expression (Krause et al., 2015).

Studies of northern latitude migratory songbirds suggest that many species have a robust adrenocortical response to acute stress upon arrival onto their breeding grounds (Reneerkens et al., 2002; Holberton and Wingfield, 2003; Meddle et al., 2003; Wingfield and Ramenofsky, 2011; Krause et al., 2015). These hormonal patterns are particularly evident in species in which males arrive first (Wingfield and Hunt, 2002). During severe weather the robust increase in corticosterone may facilitate the movement of birds away from their breeding areas to refuges further south where they will remain until the situation improves. Once weather conditions improve the birds may then return to reattempt breeding (Wingfield et al., 2004). It remains unclear how, or if, the robust stress response varies according to the latitude or location of the northern extremes of breeding grounds. Indeed, how robust the adrenocortical response to acute stress is on arrival at the breeding grounds appears to be highly variable among avian species and even among populations. For example, snow buntings (*Plectrophenax nivalis*) and Lapland longspurs (*Calcarius lapponicus*) breeding early in the season in Alaska, appear to maintain a lower adrenocortical response to stress when compared to other Arctic breeding species such as white‐crowned sparrows (*Zonotrichia leucophrys*) and American tree sparrows (*Spizella arborea*) that breed at lower latitudes in Alaska (Wingfield et al., 1995; Romero et al., 1997; Meddle et al., 2002; Holberton and Wingfield, 2003; Krause et al., 2015). However, in spite of this lower stress response, incubating Lapland longspurs on the North Slope of Alaska abandoned their nests following a 3‐day snowstorm with sub‐freezing temperatures and resumed flocking behavior typical of the non‐breeding season. This nest abandonment was accompanied by a dramatic increase in corticosterone (Astheimer et al., 1995). So clearly, if environmental conditions deteriorate for prolonged periods, there is the potential for a robust adrenocortical response to stress (Astheimer et al., 1995).

The lowest observed adrenocortical stress response in Arctic birds occurs once breeding is over and the pre‐basic feather molt begins. This is thought to be a mechanism by which detrimental effects of corticosteroids on protein synthesis and turnover are avoided during feather formation when large amounts of keratin are being produced (Romero et al., 2005). In support of this argument, experimental elevation of circulating corticosterone during molt can result in the malformation of feathers (Romero et al., 2005; DesRochers et al., 2009).

The magnitude of the adrenocortical stress response also varies with age. A “hypo‐responsive period”—where the HPA stress response is underdeveloped in young that are unable to thermoregulate and obtain their own food (i.e., altricial young)—has been well described in a number of species (Walker et al., 2005; Wada et al., 2007; Wada and Breuner, 2008; Rensel et al., 2010). A robust stress response gradually develops as chicks grow so that when chicks are ready to fledge from the nest an adult‐like stress response is typically observed.

We conducted field investigations to investigate patterns of glucocorticoid hormone release during the breeding season in both snow buntings and Lapland longspurs at the northern edge of their range in northwest Greenland. To date, our knowledge of whether Arctic breeding specialists are able to modulate their adrenocortical responses to extreme conditions upon arrival on their breeding grounds is limited. We hypothesized that these species would show a higher adrenocortical response to stress upon arrival at the northern edge of their breeding range, and, once breeding had commenced, show a decline that continues into molt. Such regional variation in the adrenocortical response to stress could be an important physiological mechanism allowing animals to breed in a range of geographical locations where the weather is extreme and unpredictable.

The modulation of the adrenocortical response to stress during the breeding season is achieved to varying degrees by changes in secretion and/or responses to adrenocorticotropic hormone (ACTH), corticotropin releasing‐factor (CRF), and arginine vasotocin (AVT) (Romero and Wingfield, 1998; Romero et al., 1998b, 1998c, 1998d). Such investigations are important to determine whether the modulation of the stress response involves changes in: (i) sensitivity of adrenocortical cells to ACTH; (ii) sensitivity of pituitary corticotrophs to CRF and AVT; (iii) release of CRF and AVT from the hypothalamus, or (iv) modification in the central nervous system prior to integration in the hypothalamus. In the present study we determined whether corticosterone levels can increase beyond those generated by the capture‐stress‐protocol and at what level of the HPA axis stress response modulation may occur. To do this we gave intravenous injections of ACTH, CRF, and AVT and quantified corticosterone release.

In addition, we compared the stress response in fledglings that were outside the nest, but still dependent on parents for food, with those fledglings that had recently become completely independent from their parents. We predicted that dependent fledglings would still have a lower stress response compared to adults as food was being provided for them. For recently independent fledglings, however, we predicted a higher stress response than adults (and dependent fledglings as well), due to their need to be obtaining food independently.

Snow buntings and Lapland longspurs are species of particular interest because both are Holarctic in distribution and breed across a wide latitudinal range throughout the Arctic. Thus, comparisons across a latitudinal gradient are possible. In the High Arctic, these birds are generally migratory. Very few spend the entire year in the north, and those that do remain are only found in lower Arctic regions. These species are also interesting because of the differences in their timing of arrival onto High Arctic breeding grounds. The snow bunting is one of the earliest songbirds to arrive in spring (Tinbergen, 1939; Irving, 1960) and because winter conditions may still be prevalent, breeding may not begin until some weeks later. In contrast, Lapland longspurs arrive later and are generally more synchronous with arrival of other migratory Arctic songbirds (SLM, BGW, and JCW, unpublished data).

## MATERIALS AND METHODS

Data collection and experimental observations were performed in and around Thule Air Force Base, Thule, Greenland (76°32′N; 68°50′W) during June and July 2001. The elevation of location of captures and observations ranged from sea level to a maximum of 300 m. All animal handling procedures were approved by the University of Washington Institutional Animal Care and Use Committee. Permits and permission for work in Greenland were obtained in association with the High Arctic Institute, Peregrine Fund, USA.

### Capture and Sampling

We captured male and female snow buntings and Lapland longspurs on their breeding grounds with mist nets, Potter traps baited with seeds, or clap nets at the nest, the latter triggered by a 5 m rope pulled by a hidden observer. Following capture, the adrenocortical response to acute capture and handling stress was assessed (Wingfield, 1994). We collected a blood sample within 3 min of capture, via puncture of the alar vein in the wing, to evaluate pre‐disturbance hormone titers, as glucocorticoid hormones levels have been shown to not significantly increase if obtained within 3 min of capture (Romero and Reed, 2005). Following baseline sampling, we placed birds into opaque cloth bags and collected subsequent blood samples at 10, 30, and 60 min. We stanched blood flood with cotton after each sampling. At each sampling time we collected approximately 30–40 μL of blood into heparinized microcapillary tubes, which were held on ice until return to the laboratory later in the day. Samples were then centrifuged at about 500*g* for 5–10 min. Resultant plasma was collected and frozen at −20°C and was kept frozen until processed for radioimmunoassay (see below).

We assigned the sex of each bird via dimorphic plumage, and determined breeding status by presence of a brood patch in females and the size of the cloacal protuberance in males. For example, a developing brood patch is indicative of nest initiation whereas a full edematous brood patch is typical of late incubation and nestling brooding (Wingfield and Farner, 1976, 1978). We assigned a fat score from the furculum and abdomen using an arbitrary scale from 0 (no visible fat) to 5 (gross bulging fat bodies; see Wingfield and Farner, 1978). Any indication of molt was recorded by the absence of primary flight feathers (remiges) and the development of replacement feathers. We fitted birds with a unique metal numbered leg band and a combination of plastic color leg bands to aid in field identification.

We assessed snow buntings for the corticosterone stress response during four stages: arrival onto breeding grounds (prior to the appearance of eggs in the nest), incubation, the feeding of nestlings, and molt. In addition, late in the season, we compared corticosterone stress responses in birds of three age classes: adults, young‐of‐the‐year receiving food from parents, or young‐of‐the‐year that were feeding independently. We assessed adult Lapland longspur males and females for their corticosterone response to stress during two stages: arrival onto breeding grounds (pre‐nesting) and post‐arrival—once birds had established a nesting site and initiated breeding activities. Finally, we compared the glucocorticoid stress responses of male snow buntings and Lapland longspurs arriving in Greenland to stress response data for arriving snow buntings and Lapland longspurs in Alaska, USA (Toolik Field Station, 67°N and Barrow, 71°N). We obtained the Alaska data from previously published work (Wingfield et al., 1994b; Astheimer et al., 1995) or data maintained in the Wingfield laboratory database.

### Function of the HPA Axis

We challenged a subset of snow bunting adults with peptide injections to assess the functionality of the HPA axis during incubation and when nestlings were being fed. To examine adrenal function, immediately after capture and initial blood sampling we injected birds (into the jugular vein) with 14 μL lactated Ringer's solution (Baxter) containing 100 IU‐kg porcine adrenocorticotropic hormone (ACTH, Sigma‐Aldrich, St. Louis, MO, USA). To examine pituitary function, we injected birds with one of three treatments including: (i) 3 μg/kg corticotropin‐releasing factor (CRF, Sigma‐Aldrich), (ii) 3 μg/kg arginine vasotocin (AVT, Sigma‐Aldrich), or (iii) 3 μg/kg each of CRF and AVT combined, all dissolved in Ringer's solution. Following all injections, we placed each bird in an opaque cloth bag for 30 min after which we collected approximately 60 μL of blood for corticosterone quantification. Due to the limited number of birds available, we chose to use as our controls in the injection study the birds caught for regular stress series, rather than injecting a suite of birds with saline. Previous studies in snow buntings have shown that hormone levels from ringer‐injected controls (Romero et al., 1998b) are essentially equivalent to non‐injected birds (Romero et al., 1998a).

### Corticosterone Radioimmunoassay

We measured corticosterone titers by radioimmunoassay after extraction from plasma with freshly re‐distilled dichloromethane. We equilibrated all samples with approximately 2,000 cpm of tritiated corticosterone as an internal standard for determination of recovery following extraction, then added corticosterone antiserum (Endocrine Sciences, Calabasas Hills, CA) to all samples. We separated bound and free hormone by addition of dextran‐coated charcoal. For details of the corticosterone assay, see Wingfield et al. (1991). Recoveries typically ranged from 50–90%. Intra‐assay variation (for a total of three assays) was 5.4%, while inter‐assay variation was 16.6%. Sensitivity of the assay was approximately 0.1 ng/ml.

### Statistical Analysis

We used SPSS for all statistical analyses, and log‐transformed all hormone data prior to calculations. We used General Linear Models (GLM) to compare baseline and integrated corticosterone (dependent variables) between sex and breeding stage (fixed effects) for snow buntings and Lapland longspurs. We also used GLM to compare differences in baseline and integrated corticosterone (dependent variables) by age of snow buntings and for the various injection treatments in snow buntings (fixed effects) as well as to compare our Greenland data with data previously collected and published in the Wingfield laboratory on birds in Alaska. We calculated integrated corticosterone (i.e., a measure of the complete corticosterone response during the capture protocol) by calculating the area under the curve using the arithmetic trapezoid rule. We used Tukey's HSD as our post‐hoc test, when required.

## RESULTS

**Figure 1:**
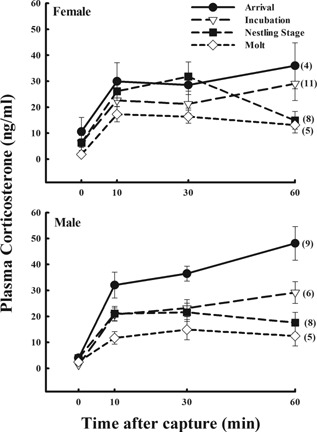
The corticosterone stress response in female and male snow buntings at Thule, Greenland. Birds were sampled during arrival onto breeding grounds, incubation, the nestling stage, and during molt. Females had higher baseline corticosterone than males (P = 0.03), but were not different in integrated corticosterone (area under the curve; P = 0.92). Across stages, baselines were again similar (P = 0.11), but integrated corticosterone during molt was lower than the three other life history stages (arrival: P < 0.001; incubation: P = 0.01; nestlings: P = 0.02). Within females, baseline and integrated corticosterone were similar across stages (P = 0.01 and P = 0.34, respectively). Baseline corticosterone was similar across stages for males (P = 0.10), but integrated corticosterone was higher at arrival as compared to nestling and molt stages (P < 0.05 and P < 0.05, respectively). Sample sizes are included in parentheses.

For snow buntings, females (n = 28) had higher baseline corticosterone than males (n = 29; F_(1,49)_ = 4.93; P = 0.03; Fig. 1) but integrated corticosterone was the same between sexes (F_(1,49)_ = 0.01; P = 0.92). When both sexes were combined, there were no significant differences in baseline corticosterone across stages (F_(3,49)_ = 2.09; P = 0.11; all stages combined; Fig. 1) but integrated corticosterone was different (F_(3,49) _= 7.67; P < 0.001), with molt being lower than the three other stages (arrival: Tukey's HSD—P < 0.001; incubation: Tukey's HSD—P = 0.01; nestlings: Tukey's HSD—P = 0.02; Fig. 1).

Within snow bunting females, neither baseline (F_(3,24) _= 1.73; P = 0.19) nor integrated corticosterone (F_(3249) _= 1.19; P = 0.34) was different across stages (Fig. 1). For males, baseline corticosterone was the same across stages (one‐way ANOVA F_(3,25)_ = 2.28; P = 0.10). However, integrated corticosterone was different across stages (F_(3,25) _= 13.85; P < 0.001) with arrival being higher than nestlings (Tukey's HSD—P < 0.05) and molt (Tukey's HSD—P < 0.05; Fig. 1).

**Figure 2:**
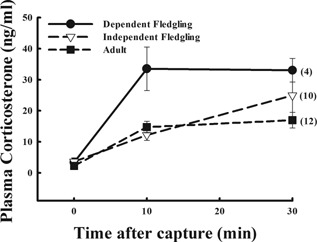
Plasma corticosterone in snow buntings of three different ages at Thule, Greenland: just‐fledged chicks still dependent on parents, independent fledglings, and adults during their molt period. Baseline corticosterone was similar across ages (P = 0.64), but integrated corticosterone was higher in dependent fledglings as compared to both independent fledglings (P* *= 0.04) or adults (P = 0.05). Sample sizes are included in parentheses.

When comparing snow buntings of different ages, we found that baseline corticosterone was not significantly different between adults (n = 12), just‐fledged chicks still dependent on parents (n = 4), and independent fledglings (n = 10; F_(2,23) _= 0.46; P = 0.64 (Fig. 2). However, integrated corticosterone was different among ages (F_(2,23) _= 3.82; P = 0.04) with just‐fledged chicks showing higher integrated corticosterone than either adults (Tukey's HSD—P = 0.04); or independent fledglings (Tukey's HSD—P = 0.05, Fig. 2).

**Figure 3:**
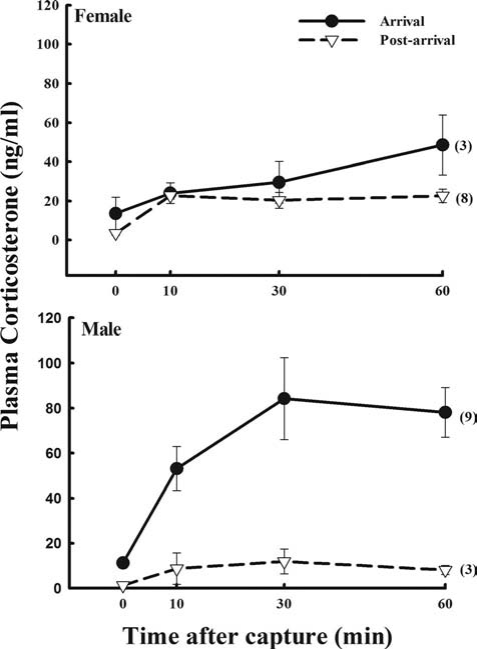
The corticosterone stress response in female and male Lapland longspurs at Thule, Greenland following capture. Baseline corticosterone was similar between the sexes (P = 0.37), but higher in arrival compared to post arrival for both sexes (P < 0.001). While integrated corticosterone (area under the curve) was not different between sexes (P = 0.77), integrated corticosterone was higher during arrival for males (P < 0.001) but females showed no differences between stages (P = 0.29). Sample sizes are given in parentheses.

For Lapland Longspurs, there were no significant differences overall between females (n = 11) and males (n = 12) in baseline corticosterone (F_(1,19) _= 0.83; P = 0.37), but baseline corticosterone was higher during arrival compared to post‐arrival for both sexes (F_(1,19)_ = 17.21; P = 0.001; Fig. 3). Integrated corticosterone was not different between sexes overall (F_(1,19)_ = 0.08; P = 0.79; Fig. 3), but both stage (F_(1,19)_ = 24.75; P < 0.001) and the interaction between sex and stage (F_(1,19)_ = 11.82; P = 0.003) were significantly different for integrated corticosterone. Post hoc analyses showed that for males, corticosterone was higher during arrival as compared to post‐arrival (F_(1,10)_ = 28.13; P < 0.001), while females showed no differences in integrated corticosterone between stages (F_(1,9)_ = 1.70; P = 0.23; Fig. 3).

**Figure 4:**
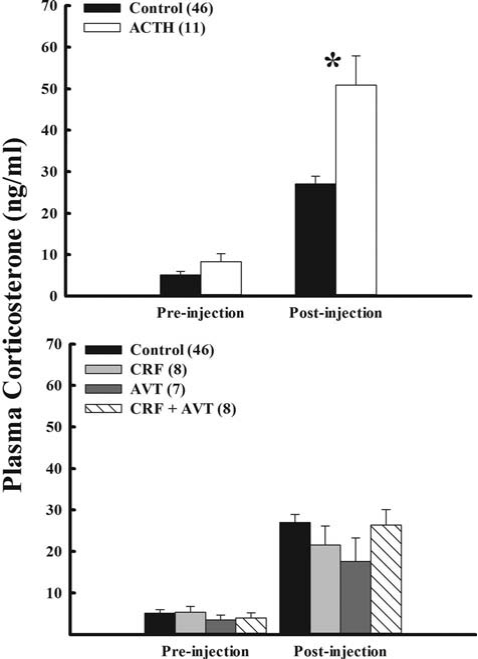
Effect of injections of adrenocorticotropin (ACTH), corticotropin releasing factor (CRF), arginine vasotocin (AVT), and combination (CRF + AVT) on plasma corticosterone in snow bunting adults in Thule, Greenland. There was no difference in pre‐injection corticosterone for ACTH‐injected and control birds (P = 0.24; top panel), but corticosterone was significantly higher in ACTH birds 30‐min post‐injection (*P = 0.01; top panel). There were no differences in corticosterone at either pre‐injection (P = 0.90) or 30‐min post‐injection between control and any of the other injection groups (CRF, AVT, CRF + AVT; P* *= 008; lower panel). Samples sizes are given in parentheses.

There was no difference in baseline corticosterone between controls (n = 46) and the ACTH treatment group (n = 11; F_(1,39)_ = 1.46; P = 0.24; Fig. 4). At 30‐min post‐injection, corticosterone was significantly higher in ACTH‐injected birds than in controls (F_(1,40)_ = 7.07; P = 0.01; Fig. 4).

There was no significant difference in baseline corticosterone among controls (n = 46) and any of the other injection groups (CRF (n = 8); AVT (n = 7), AVT + CRF (n = 8); F_(3,46)_ = 0.19; p = 0.90; Fig. 4). Similarly, corticosterone levels at 30 min were not different among treatment and control birds (F_(3,49)_ = 2.35; P = 0.08; Fig. 4).

**Figure 5:**
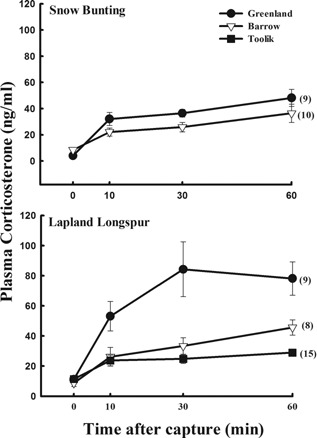
Comparison of adrenocortical responses to stress in male snow buntings and Lapland longspurs in Alaska, USA (Toolik Field Station, 67°N and Barrow, 71°N), and Thule, Greenland (76°N). For both species, the corticosterone stress response was greater in the more northerly study site—Greenland (snow buntings P = 0.05; Lapland longspurs P < 0.005). Females showed no differences in corticosterone profiles according to latitude (data not shown). (Alaska data from Wingfield et al., 1994b; Astheimer et al., 1995).

Arriving male snow buntings in Greenland had significantly higher integrated corticosterone than snow buntings that arrived on breeding grounds in Alaska (t* *= −2.12; P = 0.05; Fig. 5). As well, arriving Lapland longspurs in Greenland had significantly higher integrated corticosterone (F_(2,29)_ = 15.08; P < 0.001) compared to birds arriving in Toolik Field station, Alaska (Tukey's HSD—P < 0.001) and Barrow, Alaska (Tukey's HSD—P < 0.004; Fig. 5).

## DISCUSSION

Over a decade ago Wingfield and Sapolsky (2003) suggested that animals breeding at high latitudes, where the breeding season is extremely short, might decrease sensitivity to acute stressors to increase reproductive success, even though this may potentially reduce survival in the face of local perturbations. Evidence to date indicates that several species nesting at the northern limit of their range do indeed suppress the adrenocortical responses to a standardized stressor, but only during the later stages of breeding (Wingfield, 1994; Wingfield et al., 1995, 2004). This attenuation is thought to be regulated by a decrease in mineralocorticoid receptor expression in the hippocampus (Krause et al., 2015).

On arrival, male snow buntings and Lapland longspurs exhibited a higher glucocorticoid stress response (Figs. 1 and 3) as compared to later in the breeding season. Furthermore, this stress response is decreased in males and females of both species as nesting ensues, and in particular for Lapland longspurs, at the onset of molt (Figs. 1 and 3). This decrease as breeding season progresses has been demonstrated in populations breeding at lower latitudes in the Arctic (Wingfield et al., 1994a, 1994b). More importantly, male snow buntings and Lapland longspurs breeding in Northwest Greenland show greater adrenocortical responses to stress on arrival from migration and prior to onset of nesting compared with populations in Barrow Alaska (Fig. 5). These data are consistent with the hypothesis posited by Wingfield et al. (2004) which states that a greater adrenocortical response to stress at arrival to the breeding grounds in spring could be adaptive in allowing birds to be more reactive to variable conditions and to respond quickly and effectively if conditions are not ideal. However, once committed to nesting, these birds should then become more resistant to perturbations so as to enhance reproductive success. Additionally, the down regulation of the stress response later on in the breeding season may be a pattern in support of the brood‐value hypothesis (i.e., Heidinger et al., 2006; Lendvai et al., 2007). Specifically, as postulated by Wingfield and Sapolsky (2003), if the value in current reproduction is high, as compared to reproductive attempts in the future, then suppression of the stress response should occur, decreasing the probability of immediate nest abandonment and concomitant increased chick mortality.

Interestingly, in Lapland longspurs (Fig. 3), and in contrast to snow buntings (Fig. 1), the baseline corticosterone upon arrival was higher than at any other time in the breeding season for both males and females. Perhaps elevated corticosterone in the synchronously arriving Lapland longspurs relates to their later arrival and their need for elevated energy in order to immediately commence breeding activities. High baseline corticosterone levels have previously been reported in the red knot (*Calidris canutus islandica*), a long‐distance High Arctic migrant (Reneerkens et al., 2002).

Female birds show patterns in corticosterone secretion that are much less affected by limits to northern range, as seen in the white‐crowned sparrow, American tree sparrow, and Smith's longspur (*Calcarius pictus*) breeding at the northern edge of their ranges on the North Slope of Alaska (Holberton and Wingfield, 2003; Meddle et al., 2003; Krause et al., 2015). It is not clear why females, unlike males, do not show a higher acute stress response after arrival from migration as compared to the rest of the breeding season. In the High Arctic, females arrive within a few days of males and, thus, are exposed to the same environmental conditions, but males do establish territories on arrival and generally accompany females closely. By playing a sentry role (i.e., looking out for predators) and defending a territory, males may allow females to feed more efficiently so they accumulate greater reserves for egg laying and can spend greater time incubating (JCW, unpublished observations).

In contrast to our prediction, it was surprising to discover that snow bunting fledglings still dependent on parents for food had a high adrenocortical response to stress (Fig. 2). Moreover, independent fledglings—with no evidence of parental care or begging—had stress responses similar to adults. Previous studies in lower latitudes have shown that chicks at or near fledging have a normal, adult like stress response (Walker et al., 2005; Wada et al., 2007; Wada and Breuner, 2008; Rensel et al., 2010). Perhaps the new experience of being out of the nest, begging for food from their parents and competing for food with their siblings, greatly affected the HPA axis of these fledglings. Indeed, studies have shown that corticosterone and begging behavior can be correlated in birds (Kitaysky et al., 2001; Quillfeldt et al., 2006). We had predicted that the independent fledglings, completely independent from their parents, might have been more susceptible to the stress of having to find their own food. In reality, these independent young had a plethora of food available to them (Wingfield, unpublished data), so having learned how to obtain their own food relieved some of the need for a higher stress response elicited by the still dependent and slightly younger fledglings that are begging and competing for food with their nest‐mates.

At 30 min post‐injection, ACTH‐injected snow buntings showed significantly higher corticosterone levels than controls (Fig. 4). This indicates that the modulation of corticosterone release is occurring above the level of the adrenocortical tissue. We further tested regulatory mechanisms above the adrenal tissue by injecting snow buntings with CRF, AVT, or CRF + AVT. None of the injection treatments resulted in increased corticosterone titers as compared to controls. Thus, modulation of the adrenocortical response is most likely occurring at the level of the pituitary gland in snow buntings. This is different from results from Alaskan snow buntings where AVT successfully augmented corticosterone release (Romero et al., 1998b), presumably via increased endogenous ACTH release.

### Latitudinal Differences

A comparison of the corticosterone profiles collected here (Northwest Greenland; 76°N) with those from studies in Alaska (67–71°N) (Wingfield et al., 1994b; Astheimer et al., 1995) reveal that male snow buntings and Lapland longspurs have a markedly higher corticosterone stress response upon arrival in Greenland (Fig. 5). Hormone analyses from previous studies compared here were conducted in the Wingfield laboratory under similar protocols and conditions. Thus, we feel confident in our ability to include data from previous studies as our long‐term data set on Arctic breeding passerines makes such comparisons possible.

These findings extend our knowledge of when and how the adrenocortical response to acute stress is modulated and confirms that males of some species at the northern extremes of their breeding range enhance their stress responses at arrival. It is also likely that such flexibility in modulation of stress responses may be typical of nesting birds at their upper altitudinal range and at the leading edge of range expansion into urban areas (Bonier et al., 2007; Addis et al., 2011; Liebl and Martin, 2012). These latitudinal correlations are similar to those reported in a recent across‐avian‐species analysis of stress response vs. latitude (Jessop et al., 2013). The relationship, however, does not appear universal, as Quirici et al. (2014) found no association between latitude and the stress response in another bird species, the thorn‐tailed rayadito (*Aphrastura spinicauda*).

How an individual's stress response relates to an individual's fitness is a topic of intense interest and discussion among stress physiologists (e.g., Breuner et al., 2008; Bonier et al., 2009; Crespi et al., 2013). How large scale and abiotic environmental factors affect stress expression are also significant considerations, especially as concern over global climate change increases (Jessop et al., 2013; Wingfield, 2013). In the present studies, we were unable to measure direct fitness outcomes because the patterns of stress response expression seem to show high variability in some instances (i.e., the pattern of stress expression during the breeding chronology varies among species), while in others, there appears to be a more consistent pattern (i.e., latitudinal expression patterns). As such, further studies are required. In particular, the question of how global climate change and other anthropogenic impacts on the environment will affect the overall fitness in many different bird species is a particularly compelling question.

## References

[jez1923-bib-0001] Addis EA , Davis JE , Miner BE , Wingfield JC. 2011 Variation in circulating corticosterone levels is associated with altitudinal range expansion in a passerine bird. Oecologia 167:369–378. 21533815 10.1007/s00442-011-2001-5

[jez1923-bib-0002] Astheimer LB , Buttemer WA , Wingfield JC. 1995 Seasonal and acute changes in adrenocortical responsiveness in an Arctic‐breeding bird. Horm Behav 29:442–457. 8748507 10.1006/hbeh.1995.1276

[jez1923-bib-0003] Bonier F , Martin PR , Moore IT , Wingfield JC. 2009 Do baseline glucocorticoids predict fitness? Trends Ecol Evol 24:634–642. 19679371 10.1016/j.tree.2009.04.013

[jez1923-bib-0004] Bonier F , Martin PR , Sheldon KS , et al. 2007 Sex‐specific consequences of life in the city. Behav Ecol 18:121–129.

[jez1923-bib-0005] Breuner CW , Patterson SH , Hahn TP. 2008 In search of relationships between the acute adrenocortical response and fitness. Gen Comp Endocrinol 157:288–295. 18602555 10.1016/j.ygcen.2008.05.017

[jez1923-bib-0006] Crespi EJ , Williams TD , Jessop TS , Delepanty B. 2013 Life history and the ecology of stress: how do glucocorticoid hormones influence life‐history variation in animals? Funct Ecol 27:93–106.

[jez1923-bib-0007] DesRochers DW , Reed JM , Awerman J , et al. 2009 Exogenous and endogenous corticosterone alter feather quality. Comp Biochem Physiol A Physiol 152:46–52. 10.1016/j.cbpa.2008.08.034 18804171

[jez1923-bib-0008] Gaston AJ , Gilchrist HG , Hipfner JM. 2005 Climate change, ice conditions and reproduction in an arctic nesting marine bird: Brunnich's guillemot (*Uria lomvia* L.) J Anim Ecol 74:832–841.

[jez1923-bib-0009] Heidinger BJ , Nisbet ICT , Ketterson ED. 2006 Older parents are less responsive to a stressor in a long‐lived seabird: a mechanism for increased reproductive performance with age? Proc R Soc Lond B Biol Sci 273:2227–2231. 10.1098/rspb.2006.3557 PMC1635515 16901843

[jez1923-bib-0010] Holberton RL , Wingfield JC. 2003 Modulating the corticosterone stress response: a mechanism for balancing individual risk and reproductive success in arctic‐breeding sparrows? Auk 120:1140–1150.

[jez1923-bib-0011] Hoye TT , Post E , Meltofte H , Schmidt NM , Forchhammer MC. 2007 Rapid advancement of spring in the high Arctic. Curr Biol 17:R449–R451. 17580070 10.1016/j.cub.2007.04.047

[jez1923-bib-0012] Irving L. 1960 Birds of Anaktuvuk Pass, Kobuk, and Old Crow. Washington, DC: Smithsonian Institution p 409

[jez1923-bib-0013] Jessop TS , Woodford R , Symonds MRE. 2013 Macrostress: do large‐scale ecological patterns exist in the glucocorticoid stress response of vertebrates? Funct Ecol 27:120–130.

[jez1923-bib-0014] Kitaysky AS , Wingfield JC , Piatt JF. 2001 Corticosterone facilitates begging and affects resource allocation in the black‐legged kittiwake. Behav Ecol 12:619–625.

[jez1923-bib-0015] Krause JS , McGuigan MA , Bishop VR , Wingfield JC , Meddle SL. 2015 Decreases in mineralocorticoid but not glucocorticoid receptor mRNA expression during the short Arctic breeding season in free living gambel's white‐crowned sparrow (*Zonotrichia leucophrys gambelii*). J Neuroendocrinol 27:66–75. 25411901 10.1111/jne.12237

[jez1923-bib-0016] Lendvai AZ , Giraudeau M , Chastel O. 2007 Reproduction and modulation of the stress response: an experimental test in the house sparrow. Proc R Soc Lond B Biol Sci 274:391–397. 10.1098/rspb.2006.3735 PMC1702373 17164203

[jez1923-bib-0017] Liebl AL , Martin LB. 2012 Exploratory behaviour and stressor hyper‐responsiveness facilitate range expansion of an introduced songbird. Proc R Soc B Biol Sci 279:4375–4381. 10.1098/rspb.2012.1606 PMC3479806 22951742

[jez1923-bib-0018] Martin K , Wiebe KL. 2004 Coping mechanisms of alpine and arctic breeding birds: extreme weather and limitations to reproductive resilience. Integr Comp Biol 44:177–185. 21680497 10.1093/icb/44.2.177

[jez1923-bib-0019] Meddle SL , Owen‐Ashley NT , Richardson MI , Wingfield JC. 2003 Modulation of the hypothalamic‐pituitary‐adrenal axis of an Arctic‐breeding polygynandrous songbird, the smith's longspur, *Calcarius pictus* . Proc R Soc B Biol Sci 270:1849–1856. 10.1098/rspb.2003.2455 PMC1691444 12964988

[jez1923-bib-0020] Meddle SL , Romero LM , Astheimer LB , et al. 2002 Steroid hormone interrelationships with territorial aggression in an Arctic‐breeding songbird, gambel's white‐crowned sparrow, *Zonotrichia leucophyrs gambelii* . Horm Behav 42:212–221. 12367574 10.1006/hbeh.2002.1813

[jez1923-bib-0021] Olsson PQ , Sturm M , Racine CH , Romanovsky V , Liston GE. 2003 Five stages of the Alaskan Arctic cold season with ecosystem implications. Arctic Antarctic Alpine Res 35:74–81.

[jez1923-bib-0022] Post E , Forchhammer MC , Stenseth NC , Callaghan TV. 2001 The timing of life‐history events in a changing climate. Proc R Soc B Biol Sci 268:15–23. 10.1098/rspb.2000.1324 PMC1087595 12123293

[jez1923-bib-0023] Quillfeldt P , Masello JF , Strange IJ , Buchanan KL. 2006 Begging and provisioning of thin‐billed prions, *Pachyptila belcheri*, are related to testosterone and corticosterone. Anim Behav 71:1359–1369.

[jez1923-bib-0024] Quirici V , Venegas CI , Gonzalex‐Gomez PL , et al. 2014 Baseline corticosterone and stress response in the thorn‐tailed rayadito (*Aphrastura spinicauda*) along a latitudinal gradient. Gen Comp Endocrinol 198:39–46. 24384532 10.1016/j.ygcen.2013.12.010

[jez1923-bib-0025] Reneerkens J , Morrison RIG , Ramenofsky M , Piersma T , Wingfield JC. 2002 Baseline and stress‐induced levels of corticosterone during different life cycle substages in a shorebird on the high Arctic breeding grounds. Physiol Biochem Zool 75:200–208. 12024295 10.1086/340853

[jez1923-bib-0026] Rensel MA , Boughton RK , Schoech SJ. 2010 Development of the adrenal stress response in the Florida scrub‐jay (*Aphelocoma coerulescens*). Gen Comp Endocrinol 165:255–261. 19595691 10.1016/j.ygcen.2009.07.002

[jez1923-bib-0027] Romero LM. 2002 Seasonal changes in plasma glucocorticoid concentrations in free‐living vertebrates. Gen Comp Endocrinol 128:1–24. 12270784 10.1016/s0016-6480(02)00064-3

[jez1923-bib-0028] Romero LM , Ramenofsky M , Wingfield JC. 1997 Season and migration alters the corticosterone response to capture and handling in an arctic migrant, the white‐crowned sparrow (*Zonotrichia leucophrys gambelii*). Comp Biochem Physiol C Pharmacol Toxicol Endocrinol 116C:171–177. 9134701 10.1016/s0742-8413(96)00208-3

[jez1923-bib-0029] Romero LM , Reed J. 2005 Collecting baseline corticosterone samples in the field: is under 3 min good enough? Comp Biochem Physiol A Physiol 140:73–79. 10.1016/j.cbpb.2004.11.004 15664315

[jez1923-bib-0030] Romero LM , Reed JM , Wingfield JC. 2000 Effects of weather on corticosterone responses in wild free‐living passerine birds. Gen Comp Endocrinol 118:113–122. 10753573 10.1006/gcen.1999.7446

[jez1923-bib-0031] Romero LM , Soma KK , O'Reilly KM , Suydam R , Wingfield JC. 1998a Hormones and territorial behavior during breeding in snow buntings (*Plectrophenax nivalis*): an Arctic‐breeding songbird. Horm Behav 22:40–47. 9571012 10.1006/hbeh.1997.1432

[jez1923-bib-0032] Romero LM , Soma KK , Wingfield JC. 1998b Changes in pituitary and adrenal sensitivites allow the snow bunting (*Plectrophenax nivalis*), an Arctic‐breeding song bird, to modulate corticosterone release seasonally. J Comp Physiol B 168:353–358. 9706705 10.1007/s003600050154

[jez1923-bib-0033] Romero LM , Soma KK , Wingfield JC. 1998c Hypothalamic–pituitary–adrenal axis changes allow seasonal modulation of corticosterone in a bird. Am J Physiol 274:R1338–R1344. 9612400 10.1152/ajpregu.1998.274.5.R1338

[jez1923-bib-0034] Romero LM , Soma KK , Wingfield JC. 1998d The hypothalamus and adrenal regulate modulation of corticosterone release in redpolls (*Carduelis flammea* – an Arctic‐breeding song bird). Gen Comp Endocrinol 109:347–355. 9480742 10.1006/gcen.1997.7048

[jez1923-bib-0035] Romero LM , Strochlic D , Wingfield JC. 2005 Corticosterone inhibits feather growth: potential mechanism explaining seasonal down regulation of corticosterone during molt. Comp Biochem Physiol A Physiol 142:65–73. 10.1016/j.cbpa.2005.07.014 16125989

[jez1923-bib-0036] Romero LM , Wingfield JC. 1998 Seasonal changes in adrenal sensitivity alter corticosterone levels in Gambel's white‐crowned sparrows (*Zonotrichia leucophrys gambelii*). Comp Biochem Physiol C Pharmacol Toxicol Endocrinol 119C:31–36. 9568370 10.1016/s0742-8413(97)00167-9

[jez1923-bib-0037] Sapolsky RM , Romero LM , Minck AU. 2000 How do glucocorticoids influence stress responses? Integrating permissive, suppressive, stimulatory, and preparative actions. Endocr Rev 21:55–89. 10696570 10.1210/edrv.21.1.0389

[jez1923-bib-0038] Serreze MC , Walsh JE , Chapin FS , et al. 2000 Observational evidence of recent change in the northern high‐latitude environment. Clim Change 46:159–207.

[jez1923-bib-0039] Sturm M , McFadden JP , Liston GE , et al. 2001 Snow‐shrub interactions in arctic tundra: a hypothesis with climatic implications. J Clim 14:336–344.

[jez1923-bib-0040] Tinbergen N. 1939 The behavior of the snow bunting in spring. Trans Linn Soc N Y 5:1–94.

[jez1923-bib-0041] Wada H , Breuner CW. 2008 Transient elevation of corticosterone alters begging behavior and growth of white‐crowned sparrow nestlings. J Exp Biol 211:1696–1703. 18456897 10.1242/jeb.009191

[jez1923-bib-0042] Wada H , Hahn T , Breuner CW. 2007 Development of stress reactivity in the white‐crowned sparrow nestlings: total corticosterone response increases with age, while free corticosterone response remains low. Gen Comp Endocrinol 150:405–413. 17150217 10.1016/j.ygcen.2006.10.002

[jez1923-bib-0043] Walker BG , Wingfield JC , Boersma PD. 2005 Age and food deprivation affects expression of the glucocorticosteroid stress response in magellanic penguin (*Spheniscus magellanicus*) chicks. Physiol Biochem Zool 78:78–89. 15702466 10.1086/422769

[jez1923-bib-0044] Walsh JE , Shapiro I , Shy TL. 2005 On the variability and predictability of daily temperatures in the Arctic. Atmosphere Ocean 43:213–230.

[jez1923-bib-0045] Weatherhead E , Gearheard S , Barry RG. 2010 Changes in weather persistence: insight from Inuit knowledge. Glob Env Change Hum Policy Dimens 20:523–528.

[jez1923-bib-0046] Wingfield JC. 1994 Modulation of the adrenocortical response to stress in birds In: Davey KG, Peter RE, Tobe SS, editors. Perspectives in comparative endocrinology. Ottawa: National Research Council of Canada p 520–528.

[jez1923-bib-0047] Wingfield JC. 2013 Ecological precesses and the ecology of stress: the impacts of abiotic environmental factors. Funct Ecol 27:37–44.

[jez1923-bib-0048] Wingfield JC , Deviche P , Sharbaugh S , et al. 1994a Seasonal changes of the adrenocortical responses to stress in redpolls, *Acanthis flammea*, in alaska. J Exp Zool 270:372–380.

[jez1923-bib-0049] Wingfield JC , Farner DS. 1976 Avian endocrinology – field investigations and methods. Condor 78:570–573.

[jez1923-bib-0050] Wingfield JC , Farner DS. 1978 The endocrinology of a naturally breeding population of the white‐crowned sparrow (*Zonotrichia leucophrys pugetensis*). Physiol Zool 51:188–205.

[jez1923-bib-0051] Wingfield JC , Hegner RE , Lewis DM. 1991 Circulating levels of luteinizing hormone and steroid hormones in relation to social status in the cooperatively breeding white‐browed sparrow weaver, *Plocepasser mahali* . J Zool 225:43–58.

[jez1923-bib-0052] Wingfield JC , Hunt KE. 2002 Arctic spring: hormone–behavior interactions in a severe envrionment. Comp Biochem Physiol B Biochem Mol Biol 132:275–286. 11997229 10.1016/s1096-4959(01)00540-1

[jez1923-bib-0053] Wingfield JC , O'Reilly KM , Astheimer LB. 1995 Modulation of the adrenocortical responses to acute stress in arctic birds: a possible ecological basis. Am Zool 35:285–294.

[jez1923-bib-0054] Wingfield JC , Owen‐Ashley N , Benowitz‐Fredericks ZM , Lynn SE , Hahn TP , Wada H , Breuner C , Meddle SL , Romero LM. 2004 Arctic spring: the arrival biology of migrant birds. Acta Zool Sin 50:948–960.

[jez1923-bib-0055] Wingfield JC , Ramenofsky M. 2011 Hormone‐behavior interrelationships of birds in response to weather. Adv Study Behav 43:93–188.

[jez1923-bib-0056] Wingfield JC , Sapolsky RM. 2003 Reproduction and resistence to stress: when and how. J Neuroendocrinol 15:711–724. 12834431 10.1046/j.1365-2826.2003.01033.x

[jez1923-bib-0057] Wingfield JC , Suydam R , Hunt K. 1994b The adrenocortical responses to stress in snow buntings (*Plectrophenax nivalis*) and Lapland longspurs (*Calcarius lapponicus*) at Barrow, Alaska. Comp Biochem Physiol C Pharm Toxicol Endocrinol 108:299–306.

